# *Histoplasma* Responses to Nutritional Immunity Imposed by Macrophage Activation

**DOI:** 10.3390/jof5020045

**Published:** 2019-06-05

**Authors:** Peter J. Brechting, Chad A. Rappleye

**Affiliations:** Department of Microbiology, Ohio State University, Columbus, OH 43210, USA; brechting.1@osu.edu

**Keywords:** *Histoplasma*, macrophage, virulence, phagosome, iron, zinc, copper, immunity

## Abstract

The fungal pathogen *Histoplasma capsulatum* resides within the phagosome of host phagocytic cells. Within this intracellular compartment, *Histoplasma* yeast replication requires the acquisition of several essential nutrients, including metal ions. Recent work has shown that while iron, zinc, and copper are sufficiently abundant in resting macrophages, cytokine activation of these host cells causes restriction of these metals from intracellular yeasts as a form of nutritional immunity. Faced with limited iron availability in the phagosome following macrophage activation by IFN-γ, *Histoplasma* yeasts secrete iron-scavenging siderophores and employ multiple strategies for reduction of ferric iron to the more physiologically useful ferrous form. IFN-γ activation of macrophages also limits availability of copper in the phagosome, forcing *Histoplasma* reliance on the high affinity Ctr3 copper importer for copper acquisition. GM-CSF activation stimulates macrophage production of zinc-chelating metallothioneins and zinc transporters to sequester zinc from *Histoplasma* yeasts. In response, *Histoplasma* yeasts express the Zrt2 zinc importer. These findings highlight the dynamics of phagosomal metal ion concentrations in host-pathogen interactions and explain one mechanism by which macrophages become a less permissive environment for *Histoplasma* replication with the onset of adaptive immunity.

## 1. Introduction

The fungal pathogen *Histoplasma capsulatum* causes disease in both immunocompromised and immunocompetent individuals by subverting innate immune defenses of the mammalian host. *Histoplasma* causes thousands of hospitalizations in the United States and has been estimated to infect 25,000 to 100,000 people annually [1,2]. Infections result from inhalation of airborne fungal conidia. *Histoplasma* cells initially encounter alveolar macrophages, but host innate immune responses are insufficient in controlling *Histoplasma*. Instead, normally fungicidal macrophages provide an intracellular niche permissive for *Histoplasma* yeast replication. *Histoplasma* survival within phagocytes involves the production of virulence factors that shield fungal cells from detection and eliminate macrophage reactive oxygen species (ROS)-based defenses [3,4,5]. With activation of cell-mediated immunity, particularly CD4+ T-cell production of Th1-type cytokines, the macrophage intracellular environment becomes inhibitory to fungal growth.

In addition to macrophage strategies designed to actively kill invading microbes, phagocytes can also exploit the essentiality of elements and other nutrients by limiting their availability to pathogens. This defense mechanism against pathogenic microbes has been termed “nutritional immunity”. Nonetheless, within the phagosome, *Histoplasma* yeasts acquire sufficient nutrients to support proliferation. For example, *Histoplasma* synthesizes vitamins de novo to overcome the absence of these essential co-factors from the phagosomal compartment [6]. Trace elements present a particular challenge for intracellular *Histoplasma* yeasts as host macrophages can limit their availability and yeasts cannot simply synthesize these metals. The result is a contest between fungal metal acquisition mechanisms and macrophage efforts to restrict such from their intracellular occupants. In this review, the various ways in which *Histoplasma* yeasts acquire zinc, copper, and iron from the macrophage host are described. One aspect becoming increasingly clear is that concentrations of trace elements within the phagosome are not constant. Instead, macrophage activation is linked to enhanced limitation of trace element availability, thereby illuminating how activated macrophages, unlike resting macrophages, can restrict fungal replication.

## 2. Acquisition of Zinc

Zinc serves as an important metal cofactor for many enzymes and transcription factors in eukaryotic organisms. In mammalian cells, approximately 10% of the proteome is composed of zinc binding proteins [7], and around 8% of genes in *Saccharomyces cerevisiae* are believed to bind to zinc [8]. During normal growth of *Histoplasma* in zinc-replete media, inductively coupled plasma mass spectrometry (ICP-MS) readings determined the amount of total cellular zinc [9]. This, combined with estimates of the number of yeasts analyzed and the average volume of yeasts indicate the yeast cytoplasm has approximately 250 μM zinc. Both zinc limitation and intoxication have been implicated as host defense mechanisms against intracellular infection [9,10]. In resting macrophages, zinc concentrations are sufficiently high to support *Histoplasma* proliferation. However, during the adaptive immune response, both IFN-γ [11] and GM-CSF [12] activation of macrophages inhibits *Histoplasma* growth. This phenomenon is due in part to metal sequestration from the intracellular yeast. ICP-MS analysis of zinc concentrations in macrophages and in intracellular *Histoplasma* yeasts revealed that activation of peritoneal macrophages via GM-CSF partitioned zinc pools away from the yeasts and into the macrophage [13]. Zinc levels in macrophage whole cell lysates increased both with infection of cells by *Histoplasma* as well as with GM-CSF activation. Conversely, zinc levels in *Histoplasma* yeasts recovered from GM-CSF-treated macrophages are lower than in yeasts from resting macrophages [13]. These findings support a model whereby GM-CSF induces zinc uptake by macrophages but subsequent sequestration away from the *Histoplasma*-containing phagosome, presenting a significant hurdle to proliferation for the pathogen. This limitation of zinc from the *Histoplasma*-containing phagosome explains one way that activation of macrophages during the adaptive immune stage can control *Histoplasma* infection.

**Figure 1:**
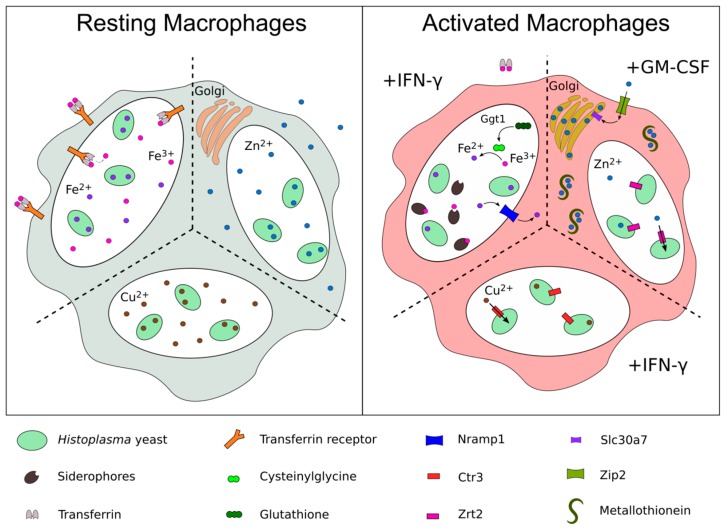
Metal acquisition mechanisms of intraphagosomal *Histoplasma* combat metal sequestration by infected macrophages triggered by inflammatory cytokines. In resting macrophages (left panel) iron, zinc, and copper levels are sufficient for *Histoplasma* growth. Upon activation (right panel), infected macrophages attempt to deprive *Histoplasma* of essential metals through divalent cation exporters (e.g., Nramp1 and Slc30a7), zinc chelating metallothioneins, and as-yet undefined mechanisms. Under these metal-limited conditions, *Histoplasma* in turn upregulates the copper and zinc importers, Ctr3 and Zrt2, respectively. Additionally, *Histoplasma* secretes siderophores to scavenge iron, as well as the Ggt1 protein, which catalyzes a ferric reduction pathway, providing ferrous iron for the yeast.

Gene expression changes following GM-CSF treatment provide some clues as to the mechanism by which zinc is sequestered from intramacrophage *Histoplasma* yeasts. GM-CSF treatment correlates with upregulation of metallothioneins (MTs) [13], host proteins capable of binding up to seven zinc molecules at picomolar concentrations [14]. Mice express 4 different MTs, while humans have 17 copies [15]. In murine macrophages, two isoforms, Mt1 and Mt2, are primarily responsible for sequestering zinc from *Histoplasma* [13]. Mt1 and Mt2 are highly expressed by the macrophage following infection by *Histoplasma* and activation by GM-CSF [13] (Figure 1). Confirming their putative involvement during *Histoplasma* infection, silencing of Mt1 and Mt2 expression prevented the GM-CSF-triggered decrease in *Histoplasma* yeast zinc concentrations [13]. GM-CSF signals are mediated through a STAT3/STAT5 dependent pathway [16]. Silencing of *STAT3* and *STAT5* decreased zinc bound to MTs, [13] positioning STAT3/STAT5 as the signaling pathway between GM-CSF signals and the resultant zinc sequestration from *Histoplasma* yeasts.

In addition to MT-binding of labile cytosolic zinc, activated macrophages store zinc in intracellular compartments to make this metal inaccessible to *Histoplasma* in the phagosome. Spatial resolution of intracellular zinc pools using the fluorescent zinc probe Zinpyr-1 showed that GM-CSF activation of macrophages leads to zinc transport and storage in the Golgi apparatus [13]. This result suggests an important role for host zinc importers (ZIPs) and exporters (ZNTs) during macrophage activation. Macrophage treatment with GM-CSF upregulated several zinc transporters in the macrophage, with the zinc importer, ZIP2, showing the largest increase in transcription [13]. While ZIP2 appears to be the primary method for the uptake of extracellular zinc in a classical macrophage activation response [7], siRNA-mediated silencing of *ZIP2* did not affect intracellular zinc levels in *Histoplasma* during infection, leaving its role in zinc nutritional immunity against *Histoplasma* unresolved. Slc30a4 and Slc30a7, two zinc-exporting ZNTs, were also upregulated by GM-CSF stimulation [13], and members of this protein family have been reported to shuttle zinc into the Golgi apparatus [17], suggesting that they may be responsible for importing labile cytosolic zinc into the organelle.

In addition to nutritional immunity, zinc import by the host cell is related to the production of anti-microbial reactive oxygen species (ROS). GM-CSF increases the oxidative burst of phagocytes upon activation [18]. When GM-CSF-activated macrophages are grown in low zinc media and infected with *Histoplasma*, they exhibit increased ROS production and inhibit yeast growth more efficiently [13]. This inhibition of intracellular *Histoplasma* growth was dependent on macrophage ROS-production and was reversed by addition of ZnSO_4_ to the growth media [13]. Depletion of host Mt1 and Mt2 diminished GM-CSF-triggered ROS production, linking the labile zinc pool to enhanced oxidative burst and increased pathogen clearance [13]. Interestingly, the addition of zinc to the media improved *Histoplasma* survival against ROS-producing macrophages as well as an in vitro ROS generating system [13]. *Histoplasma*’s Sod3, a Cu/Zn-type superoxide dismutase [5], may explain in part *Histoplasma*’s greater survival when zinc is abundant. Together this, suggests that the inhibition of *Histoplasma* in low zinc results from a combination of enhanced macrophage ROS production as well as decreased ROS resistance of the yeasts.

The effect of the alternative cytokine IL-4 on both zinc availability and *Histoplasma* intracellular proliferation provides further evidence that zinc sequestration is an effective host defense against *Histoplasma* yeast. Macrophage activation by the cytokine IL-4 promotes differentiation into an M2 phenotype, which can prevent effective clearance of intracellular pathogens, including *Histoplasma* [19,20,21]. *Histoplasma* contains higher levels of zinc during infection of M2 macrophages than in resting macrophages [7]. Additionally, isotopic labeling of zinc revealed that M2 macrophages increase the labile zinc pool by importing zinc from the extracellular milieu, and that this zinc becomes available to *Histoplasma* in the phagosome. This effect was reversed by silencing of a metallothionein, Mt3, or a phagolysosome-localized zinc exporter, Slc30a4 [7]. Treatment with IL-4 during a mouse lung infection increases fungal burden, but this effect was mitigated in Mt3-silenced macrophages [7]. These results demonstrate that increased zinc availability for *Histoplasma* in M2-differentiated macrophages correlates with increased intracellular proliferation, highlighting the importance of nutritional immunity during infection.

To combat the host limitation of available zinc in the phagosome, yeasts express high affinity zinc transporters. In response to zinc deprivation, *Histoplasma* yeasts increased expression of the Zrt2 zinc transporter [22]. *Histoplasma* Zrt2 has sequence similarity to the characterized zinc transporter Zrt1 of *Saccharomyces cerevisiae* and ectopic expression of *Histoplasma* Zrt2 functionally complements Zrt1 deficiency in *S. cerevisiae*, confirming the function of Zrt2 as a zinc transporter [22]. RNAi-based knockdown of *ZRT2* impaired growth of *Histoplasma* yeasts in zinc-limited media consistent with its role in zinc import into yeast cells [22]. In an in vivo model of respiratory and disseminated histoplasmosis, depletion of the Zrt2 transporter attenuated *Histoplasma* virulence. Additionally, mice survived a normally lethal infection when *Histoplasma* yeasts lacked Zrt2 [22]. Importantly, attenuation of Zrt2-depleted *Histoplasma* yeasts occurred only after three days of infection, showingthat zinc is sufficiently available in resting macrophages, but becomes restricted with the development of the immune response that includes GM-CSF [22].

Together, these data on both the host and pathogen sides demonstrate that GM-CSF activity on macrophages restricts zinc availability to intracellular *Histoplasma* yeasts. The full virulence of Zrt2-deficient *Histoplasma* in resting macrophages indicates sufficient levels of phagosomal zinc. However, with the production of GM-CSF, zinc nutritional immunity provides one mechanism of controlling fungal growth.

## 3. Acquisition of Copper

Copper is an important metal cofactor for enzymes involved in a variety of physiological pathways essential for pathogens and non-pathogenic organisms. Two studies have quantified total copper content of *Histoplasma* yeasts [9,23]. These values and the average volume of yeasts estimates the cellular copper levels range from 20–80 µM during in vitro growth in copper-containing media, which is similar to estimates in other fungal cells [24]. This copper satisfies the nutritional requirement for *Histoplasma*, and also contributes to virulence-specific functions such as the Sod3 extracellular Cu/Zn superoxide dismutase enzyme, which detoxifies macrophage-derived ROS [5]. The presence of a copper chelator is sufficient to abolish Sod3 activity in vitro [5], demonstrating the importance for copper in virulence-related functions. In other fungal pathogens, melanin is synthesized though pathways involving copper cofactor-containing enzymes [25,26], and melanin can contribute to pathogenesis of *Cryptococcus neoformans* and *Paracoccidioides brasiliensis* [27,28]. *Histoplasma* can synthesize melanin-like compounds during infection [29], although a direct role for melanin in the virulence of *Histoplasma* has not yet been demonstrated.

Phagocytes can employ strategies of both copper excess as well as copper restriction for inhibition of microbial growth. Studies with intracellular bacterial pathogens indicate that the copper concentration in bacteria-containing compartments is toxic to most bacteria since only bacteria with mechanisms to cope with high copper survive. Viability and virulence of *Salmonella enterica* serovar Typhimurium was significantly decreased without the function of CopA and GolT bacterial copper export proteins [30]. This phenotype was rescued by elimination of the ATP7A mammalian copper transporter [30] which imports copper from the cytosol into the phagosome [31]. Similarly, *Mycobacterium tuberculosis* intracellular survival depended on copper exporters and the RicR regulon to resist the high copper environment inside the phagosome [32,33]. Instead of copper exporters, the fungal pathogen *Cryptococcus neoformans* uses copper-sequestering metallothioneins to reduce the toxicity of high copper environments during lung infection [34]. Together these studies suggest macrophage control of intracellular pathogens employs copper toxicity.

Consistent with this, the *Histoplasma*-containing phagosome within unactivated phagocytes is characterized by elevated copper levels. The *Histoplasma CTR3* gene encodes a copper transporter whose expression is low in high copper environments but is induced as copper becomes limiting [23]. Transcriptional fusions between the *CTR3* promoter and green fluorescent protein provided a fluorescent sensor of copper levels, which showed available copper in the Histoplasma-containing phagosome of primary macrophages was sufficiently high to prevent induction of *CTR3* expression [23]. Importantly, copper was also relatively high (estimated to be at least 0.3 µM) in resting alveolar macrophages, the initial host cell encountered by *Histoplasma* during infection. A similar approach with copper-regulated promoters in *Cryptococcus* yeasts showed that the *Cryptococcus*-containing phagosome of RAW264.7 and J774.1 macrophages has high copper [35,36]. Given *Histoplasma*’s tolerance to high copper levels (up to mM concentrations) [23], the high copper environment of the unactivated macrophage phagosomes does not inhibit *Histoplasma* growth and proliferation.

In contrast to resting macrophages, activated macrophages restrict copper from *Histoplasma* yeast within the phagosome. The Ctr3 transporter is a copper importer that is necessary for *Histoplasma* growth when copper is limited [23]. While Ctr3 was dispensable for *Histoplasma* proliferation within unactivated macrophages, growth within cytokine-activated macrophages required Ctr3, consistent with activation converting the phagosome to a copper-limited environment [23]. Ctr3-deficient *Histoplasma* yeast were fully virulent though the innate immune phase, but are attenuated in mice following activation of adaptive immune responses. A survey of macrophage-activating cytokines identified IFN-γ as both necessary and sufficient for regulating the phagosome switch to a low copper state [23]. *Histoplasma* yeasts within activated macrophages or macrophages collected from mice after the onset of adaptive immunity showed increased expression of the *CTR3* gene further substantiating the cytokine-induced conversion to a copper-limited phagosome [23].

Together, the genetic and *CTR3* transcriptional evidence indicate that phagosomal copper levels differ between unactivated and activated macrophages. Initially, phagosomes contain higher levels of copper in efforts to inhibit microbial pathogens through copper toxicity. For *Histoplasma* infections, this proves ineffective, and the pathogen grows unhindered. Adaptive immunity and the corresponding production of IFN-γ induce changes in the phagosome, through currently undefined mechanisms, to restrict copper from *Histoplasma* in efforts to starve fungal cells. *Histoplasma* yeasts combat this deprivation of phagosomal copper with expression of the Ctr3 transporter.

## 4. Acquisition of Iron

The necessity of iron as a nutritional requirement has been well documented for many pathogens [37,38], making iron limitation a key method for restricting the growth of microbial invaders. Total cellular iron concentrations fluctuate depending on the environmental iron concentration [39]. During in vitro growth in iron-containing media, *Histoplasma* yeasts accumulate iron to concentrations around 2 mM based on ICP-MS analysis [9,23] and the average cellular volume of yeasts. Intracellular *Histoplasma* is sensitive to iron limitation, as the addition of a variety of iron chelators inhibited *Histoplasma* growth in macrophages [11,40,41]. Free iron levels are kept low in the human body largely due to binding by transferrin and other iron chelators. This sequestration of iron helps to control microbial proliferation extracellularly, but may actually increase access to iron for intracellular pathogens such as *Histoplasma* due to trafficking of iron-loaded holo-transferrin to phagosomal compartments where the acidic environment of the vesicles causes release of iron into the lumen [42]. However, after macrophage activation by proinflammatory cytokines such as IFN-γ, transferrin receptors that capture extracellular iron-loaded transferrin are downregulated, and iron can be transported out of the phagosome by the iron exporter, Nramp1 [37,43]. In addition, increased cytosolic iron in macrophages can increase transcription of the nitric oxide synthase (NOS) gene [44,45], which generates antimicrobial nitric oxide (NO) [46]. This suggests that a dual effect of iron deprivation from the phagosome and increased NOS function may work in tandem to kill intracellular pathogens [47]. Neither Nramp1 nor the effects of iron on the production of NO have been determined specifically for *Histoplasma* infections. Iron deprivation does enhance clearance of *Histoplasma* during infection in mice; IFN-γ activation facilitated clearance of infection in mouse peritoneal macrophages, and this effect was reversible upon supplementation with holo-transferrin [11,48], indicating that iron limitation is an important aspect of the host response to *Histoplasma*. There have been conflicting reports as to whether IFN-γ activation restricts fungal growth in human macrophages, and dedicated iron restriction has not been directly implicated as a defense strategy of human macrophages [12,49]. However, disruption of iron acquisition mechanisms in the yeasts attenuated growth in human phagocytes, demonstrating that iron acquisition by *Histoplasma* is necessary for successful infection [40,50]. Given the importance of iron acquisition to intraphagosomal growth, *Histoplasma* employs multiple mechanisms of iron scavenging as evidenced by the large transcriptional shift that occurs in response to iron starvation [51]. 

The secretion of low molecular weight iron chelating siderophores is a method of iron acquisition that is well documented in many microbial species, including *Histoplasma* [52,53,54]. When grown in low iron conditions, *Histoplasma* secreted the hydroxamate-type siderophores dimerum acid, coprogen B, and fusarinine [55]. The *SID1* gene, which encodes, the L-ornithine monooxygenase that catalyzes the first step of dedicated hydroxamate siderophore biosynthesis, is part of a 327-gene regulon that was controlled by iron availability [56]. A GATA-type transcription factor, Sre1, negatively regulated *SID1* and six other putative siderophore biosynthesis and utilization genes as well as genes encoding ferric reductase pathways [51]. Loss of Sid1 function, either through gene deletion or RNAi-mediated knockdown of the *SID1* gene, prevented siderophore production impairing growth of *Histoplasma* in low iron media and attenuating *Histoplasma* growth in macrophages [50,56]. The reversibility of these phenotypes upon addition of excess FeSO_4_ indicates the attenuation was due to the inability to scavenge rare iron [50,56]. Virulence of siderophore-deficient *Histoplasma* yeasts is attenuated, particularly following activation of adaptive immunity [56], again implicating cytokine-induced nutritional immunity as a host defense mechanism.

In addition to the acquisition of iron via siderophores, *Histoplasma* can acquire biologically relevant ferrous iron through reduction of ferric iron. Three distinct iron reduction mechanisms have been identified: a cell surface iron-reducing agent, a non-proteinaceous low molecular weight secreted reducing agent, and secretion of a γ-glutamyltransferase protein, Ggt1, which can reduce ferric iron in a glutathione (GSH)-dependent manner [57,58]. Little is known about the cell surface and non-proteinaceous iron reduction mechanisms and no pathogenesis relevance has been demonstrated for either of these pathways [58]. Ggt1 can catalyze the transfer of a γ-glutamyl residue from GSH to a variety of different acceptors [59] leaving cysteinylglycine, which in turn served as a potent extracellular ferric reductant [57]. While GSH is capable of reducing iron, cysteinylglycine showed over 100-fold increased reduction potential [57]. Interestingly, GSH is highly abundant in macrophages (up to mM levels), especially in the lungs, though no direct measurement of GSH in the phagosomal compartment has been reported [60,61]. Functionally, *Histoplasma* Ggt1 is required for maximal macrophage killing by *Histoplasma* yeasts [57], suggesting a role for iron reduction in virulence. However, no in vivo functional tests have been done, leaving the relevance of this iron reduction system to *Histoplasma* pathogenesis unknown. In addition, the genomes of several strains of *Histoplasma* (Panama, Latin America, and NAm1 lineages), but not the North American type 2 (NAm2) strains, encode homologs of the Fet3 and Ftr1 ferric reduction and import system which has been characterized in *Saccharomyces cerevisiae* [50,62]. However, because this putative iron acquisition system has not been depleted or overexpressed in the relevant backgrounds and examined during infection, the role of Fet3-Ftr1 in *Histoplasma* pathogenesis remains unknown.

In both human and murine macrophages, the luminal pH is near 6.5 in the *Histoplasma*-containing phagosome [63,64] which releases one of the two iron atoms bound to transferrin. This liberated iron could provide for intracellular *Histoplasma*’s nutritional needs [65]. In support of this, treatment with chloroquine, which raises the pH of the phagosome causing transferrin to remain in the fully saturated form, attenuated growth of *Histoplasma* in macrophages as well as reduced fungal burdens after 14 days in vivo [40]. Growth in macrophages was rescued by the addition of FeNTA, which provides a source of iron independent of pH [40]. However, a different study showed that a siderophore structurally similar to native *Histoplasma* dimeric acid could directly steal iron from holo-transferrin, and that *Histoplasma* could grow at neutral pH with holo-transferrin as the only iron source [41], together suggesting pH-dependent release of iron from transferrin may not be necessary. In addition, *Histoplasma* could bind hemin to its cell surface, and could use hemin as a sole iron source, which has led to speculation that hemin may serve as another source of nutritional iron in the host. However, this hypothesis has not been examined in macrophages [66].

Whether these multiple sources and multiple mechanisms for acquisition of ferrous iron are redundant or combine to efficiently provide intraphagosomal yeasts with essential iron remains to be fully determined. In addition, studies have connected the *Histoplasma* Vma1 vacuolar ATPase with iron homeostasis. A strain lacking Vma1 was unable to grow in low-iron media and exhibited attenuated virulence in macrophages and in vivo in mice [67]. The addition of FeNTA or holo-transferrin restored some fungal growth in a macrophage model. This suggests that the vacuolar ATPase of *Histoplasma* plays some undetermined role in iron acquisition, but the lack of full rescue of growth may indicate that Vma1 affects additional processes unrelated to iron which are important for full virulence [67].

It is apparent that the host attempts to sequester iron during *Histoplasma* infection and, as with zinc and copper, activation of adaptive immunity enhances the macrophage restriction of iron from the yeasts [37,43,56]. Consequently, *Histoplasma* yeasts devote considerable efforts to scavenging this metal from the phagosomal environment to satisfy its nutritional requirement.

## 5. Conclusions

As a primary pathogen, *Histoplasma* uses multiple mechanisms to overcome the host’s immune response. This is most evident during the early immune response, when *Histoplasma* yeast effectively neutralize innate immune defenses and proliferate largely unhindered. There is now substantial evidence that the adaptive immune response presents significant challenges to intraphagosomal *Histoplasma* yeasts through metal-related nutritional immunity. The availability of three major biologically-essential metals (zinc, copper, and iron) becomes even more restricted when macrophages are activated by proinflammatory cytokines (summarized in Figure 1). Although the ability of IFN-γ and GM-CSF to limit *Histoplasma*’s access to metals has been demonstrated, the potential role of additional cytokines, the levels required for induction of nutritional immunity, and their sources are only partially understood, and could serve as the subject of continuing research. Nonetheless, the temporal dynamics and role of T-helper cells as major producers of IFN-γ strongly implicate CD4+ cells as central players in this switch. While many metal concentrations have been studied in whole cells (i.e., the macrophage), the *Histoplasma*-containing phagosome is actually the relevant compartment during infection, placing a premium on methodology that can quantify metal availability at the subcellular level. In this regard, fluorescent transcriptional reporters that indicate changes not in the absolute metal concentrations, but in their relative availability within the pathogen-containing compartment, are instructive. Genetic studies with yeast lacking specific metal acquisition mechanisms further confirm the metal concentration dynamics with regards to *Histoplasma* infection and define the numerous methods by which *Histoplasma* effectively scavenges limited metals from the host. As a dimorphic fungus with distinct saprobic and parasitic lifestyles, the question of how metal concentrations might change as *Histoplasma* moves from the soil environment to the mammalian host macrophage is an intriguing question. The finding that Ctr3 expression is partially controlled by differentiation into yeasts (Ray, unpublished data) suggests that available metal concentrations may substantially differ between the two environments. Alternatively, scarcity of available metals in the soil environment may have facilitated the evolution of mechanisms with which *Histoplasma* can combat metal nutritional immunity in the host (e.g., siderophore production and ferric reduction mechanisms). Regardless, the studies highlighted in this review that show the dynamics of metal levels in the mammalian host and the responses of *Histoplasma* shed light on conditions that may be experienced by other phagosomal pathogens and highlight the potential for therapeutic interventions that enhance metal sequestration or impair pathogen mechanisms that combat nutritional immunity.
